# Characterization of the microRNAome in Porcine Reproductive and Respiratory Syndrome Virus Infected Macrophages

**DOI:** 10.1371/journal.pone.0082054

**Published:** 2013-12-05

**Authors:** Julie A. Hicks, Dongwan Yoo, Hsiao-Ching Liu

**Affiliations:** 1 Department of Animal Science, North Carolina State University, Raleigh, North Carolina, United States of America; 2 Department of Pathobiology, University of Illinois at Urbana-Champaign, Urbana, Illinois, United States of America; University of Hong Kong, China

## Abstract

Porcine Reproductive and Respiratory Syndrome Virus (PRRSV), a member of the arterivirus family, is the causative agent of Porcine Reproductive and Respiratory Syndrome (PRRS). PRRS is characterized by late term abortions and respiratory disease, particularly in young pigs. Small regulatory RNAs termed microRNA (miRNA) are associated with gene regulation at the post-transcriptional level. MiRNAs are known to play many diverse and complex roles in viral infections. To discover the impact of PRRSV infections on the cellular miRNAome, Illumina deep sequencing was used to construct small RNA expression profiles from *in vitro* cultured PRRSV-infected porcine alveolar macrophages (PAMs). A total of forty cellular miRNAs were significantly differentially expressed within the first 48 hours post infection (hpi). The expression of six miRNAs, *miR-30a-3p*, *miR-132*, miR-27b*, *miR-29b*, *miR-146a* and *miR-9-2*, were altered at more than one time point. Target gene identification suggests that these miRNAs are involved in regulating immune signaling pathways, cytokine, and transcription factor production. The most highly repressed miRNA at 24 hpi was *miR-147*. A *miR-147* mimic was utilized to maintain *miR-147* levels in PRRSV-infected PAMs. PRRSV replication was negatively impacted by high levels of *miR-147*. Whether down-regulation of *miR-147* is directly induced by PRRSV or if it is part of the cellular response and PRRSV indirectly benefits remains to be determined. No evidence could be found of PRRSV-encoded miRNAs. Overall, the present study has revealed that a large and diverse group of miRNAs are expressed in swine alveolar macrophages and that the expression of a subset of these miRNAs is altered in PRRSV infected macrophages.

## Introduction

Porcine Reproductive and Respiratory Syndrome Virus (PRRSV) is the causative agent of PRRS, which is characterized by episodes of reproductive failure in pregnant sows and respiratory illness, particularly in young pigs. PRRSV is a major concern for the swine industry with PRRS-associated costs of hundreds of millions of dollars annually for the US swine industry alone [1]. Thus it is imperative to determine the consequences of PRRSV infections on cellular gene regulatory networks. It has been well established that small regulatory RNAs, in particular microRNA (miRNA) play many complex roles during viral infections.

MiRNAs are small non-coding RNA which mainly function in post-transcriptional gene regulation. MiRNAs recognize partial complementary sites within a targeted mRNA, typically within the 3′ untranslated region (UTR) and reduce subsequent protein production. Numerous eukaryotic miRNAs have been identified, many of which are associated with pathogenic diseases and host immunity. There are currently several hypotheses regarding the role of miRNA-mediated gene regulation in the immune system (reviewed by [2]). Firstly, miRNA targeting reduces protein expression but does not completely abate it. Therefore, miRNA regulation may serve to tightly regulate the cellular response to pathogenic infections. Secondly, as miRNAs function post-transcriptionally they can alter gene expression patterns much faster than transcriptional regulators in response to infection. Finally, miRNA-mediated regulation affects nascent protein production, not the function of already produced protein and therefore may serve as part of negative feedback regulation.

A few swine viruses have been shown to impact the miRNAome, including pseudorabies virus and influenza virus. Infection of porcine dendritic cells (DCs) with the alphaherpesvirus pseudorabies virus alters the expression of several cellular miRNAs, including miR-27b*, *miR-29a* and *miR-30e-3p* [3]. *In silico* target analysis of these differentially expressed cellular miRNAs suggests that they may be involved in viral latency. Swine influenza virus (H1N1) has also been shown to impact miRNA expression [4]. *In vitro* swine influenza infection of A549 cells (a human alveolar basal epithelial cell line) results in the up-regulation of 39 cellular miRNAs and the down-regulation of 13. Target gene analysis indicates that these differentially expressed miRNAs possess a variety of functions including regulation of the immune system and cell proliferation. Another study found potential binding sites for 36 porcine miRNAs in swine influenza encoded genes [5]. Of these, targeting by the three miRNAs *miR-124a*, *miR-136*, and *miR-145* appears to have been evolutionarily conserved, suggesting that these miRNA: viral gene interactions are an essential part of influenza pathogenesis.

Although to date little is known about the involvement of miRNAs in PRRSV infections, two recent studies suggest a role for miRNA-mediated gene regulation in PRRSV pathogenesis [6], [7]. Introduction of *miR-181* mimics into PAMs greatly reduced PRRSV replication *in vitro*. This reduction was attributed to targeting of PRRSV RNA via a *miR-181* seed match located downstream of ORF4 [6]. The cellular miRNA *miR-125b* was found to be down-regulated in PRRSV-infected cells [7]. Introduction of a *miR-125b* mimic into MARC-145 cells, a PRRSV permissive cell line, resulted in reduced PRRSV replication [7]. This reduction in viral replication was attributed to the modulation of NFκB expression by *miR-125b*. *MiR-125b* targets κB-RAS2 which serves as a negative regulator of NFκB. Thus it was speculated that PRRSV infections induce the down-regulation of *miR-125b*, which subsequently results in increased κB-RAS2 expression and ultimately reduced NFκB expression. This may serve as part of the immune evasion strategy of PRRSV by limiting the NFκB signaling response to infection. In the present study to identify alterations in miRNA expression associated with PRRSV, PAMs were infected with PRRSV strain VR-2332 and small RNA expression profiles were generated at 12, 24, and 48 hours post-infection (hpi). Overall, forty cellular miRNAs were differentially expressed in at least one time point in PRRSV-infected PAMs. However, no evidence could be found of PRRSV-encoded miRNAs. Target gene/pathway prediction and validation, using a retroviral-based system, indicates that cellular miRNAs impacted by PRRSV infection are likely involved in regulating the inflammatory response, cytokine signaling, toll-like receptor signaling, and calcium metabolism. The most repressed miRNA at 24 hpi was *miR-147*. Forced over-expression of *miR-147* in PRRSV-infected PAMs had a negative impact on viral replication. Whether down-regulation of *miR-147* is directly induced by PRRSV or if it is part of the cellular response and PRRSV indirectly benefits remains to be determined.

## Materials and Methods

### Cells and virus

Primary alveolar macrophages (PAMs) were collected via bronchoalveolar lavage from three 7-week old pigs, all animal protocols were approved by the North Carolina State University Institutional Animal Care and Use Committee. PAMs were cultured in RPMI 1640 supplemented with L-glutamine, penicillin (100 U/ml), streptomycin (100 µg/ml) and fungizone (4 µg/ml) and 10% FBS. PRRSV strain VR-2332 was used for all experiments. Virus titers were determined using the Reed-Muench method and immunoflouresence staining of the viral nucleocapsid protein (SDOW-17, Rural Technologies, INC).

DF1 cells, a chicken embryonic fibroblast cell line (obtained from ATCC), were transfected with RCAS-*miRNA* vectors using Fugene 6 according to the manufacturer's instructions (Promega) and maintained in RPMI 1640 with 1% FBS, L-glutamine, penicillin (100 U/ml), streptomycin (100 µg/ml), and fungizone (4 µg/ml), at 37°C with 5% CO_2_. Infection was confirmed and the viruses were tittered by immunofluorescence staining with the mouse monoclonal 3C2 antibody against the viral gag protein (Developmental Studies Hybridoma Bank at University of Iowa) and FITC-conjugated goat anti mouse IgG (Invitrogen). Ectopic expression of miRNA expression was validated using real-time PCR.

### Illumina small RNA sequencing

A total of 2×10^7^ PAMs per plate were maintained for 24 hours at 37°C with 5% CO_2_ and then either mock infected or infected with PRRSV strain VR-2332 at an M.O.I. of ten in duplicate and maintained at 4°C for 4 hrs. Cells were washed with 1XPBS and fresh media was added and then maintained at 37°C with 5% CO_2_. Cells were collected in Tri-Reagent (Sigma) at 0 hours post infection (hpi), 12 hpi, 24 hpi, or 48 hpi. Duplicate plates were pooled, and total RNA was purified following the manufacturer's instructions with the exception that RNA was precipitated overnight at −20°C. RNA was quantified using a nanodrop ND-1000 spectrophotometer and quality was assessed using agarose gel electrophoresis. Small RNAs were enriched using a miRVana miRNA isolation kit (Ambion) and samples were subjected to on column DNase treatment. Small RNA populations were quantified using a nanodrop ND-1000 spectrophotometer.

Small RNA libraries were generated using a TruSeq Small RNA sample preparation kit (Illumina) and barcode indices RPI1-RPI21 following the manufacturer's instructions. For each library 1 µg of enriched small RNAs was used. The quality and quantity of the libraries were assessed on an Agilent Technologies 2100 Bioanalyzer using a high sensitivity DNA chip. Each library was diluted to 10 nM using 10 mM Tris-HCl (pH 8.5) and then 4 µl of each library was pooled. Pooled DNA was sequenced on a single lane of an Illumina Genome Analyzer IIx (GAIIx) (NCSU Genomic Sciences Laboratory).

### Data analysis

All FASTQ sequencing files have been deposited to the NIH Short Read Archive (accession numbers SRR1005736, SRR1005738- SRR1005748, SRR1005789, SRR1005790, SRR1005837- SRR1005843). Reads were sorted according to the barcode index and adapter sequences were trimmed. Only high quality reads (overall Phred ≥20) were selected.

Identical sequences in each library were grouped using the GALAXY bioinformatics suite (https://main.g2.bx.psu.edu/). Known porcine miRNAs, homologous miRNAs from other species not yet present in the pig database in miRBase (http://www.mirbase.org), and other types of RNA were identified using the DSAP small RNA sequence analysis pipeline (MiRNA read numbers were normalized using counts per million (CPM). Differentially expressed miRNAs were determined using the Cox-Reid common dispersion analysis and the negative binomial general linear model (NB GLM) likelihood ratio test [8]. Only miRNAs represented by >20 reads were selected for differential expression testing. Pairwise differential expression comparisons were made between time-matched mock and VR-2332 infected PAMs. Values are presented as log fold differences.

### miRNA target prediction analysis

Potential target genes for selected significantly differentially expressed miRNA were identified using the *Sus scrofa* Unigene database (NCBI) and the miRanda algorithm (version 3.3; http://www.microrna.org) with the following parameter settings: score threshold >130 and free energy threshold <−16 kCal/mol. The list of potential target genes was further filtered using the following higher stringency methods: (1) a match between nucleotides 2–8 of the miRNA with the target sequence or (2) a match between nucleotides 2–7 and 13–16 of the miRNA with the target sequence (G:U wobble tolerance) and (3) miRNA binding sites must lie within the 3′UTR. For each potential target gene, the 3′UTR flanking the miRNA binding site(s) were PCR amplified from pig genomic DNA using gene specific primers (Table S1). Each PCR product was cloned into the 3′ UTR of the *Renilla* luciferase reporter gene in the psiCHECK-2 vector (Promega) using the NotI and XhoI restriction sites. The psiCHECK-2 vector contains both the Renilla luciferase reporter gene to monitor small RNA targeting as well as the independent firefly luciferase reporter gene to account for any differences in transfection efficiency.

### Construction of RCAS expressing pig miRNAs vectors

The RCASBP(A)-miR vector previously described by Chen *et al.* (2008) [9] was utilized for ectopic miRNA expression or for a scrambled control sequence (SC). This system utilizes gateway cloning technology to insert and express a miRNA hairpin from the retroviral LTR region. The scrambled control sequence is expressed in the context of the *miR-30a* hairpin. MiRNA hairpin primers were designed based on the porcine precursor sequences for each miRNA. PAGE-purified forward and reverse primers (Invitrogen) (Table S1) were mixed at a final concentration of 1 µM, denatured at 95°C for 20 sec and annealed at RT. The DNA fragment was then cloned into the pENTR3C-miR-SphNgo vector at the SphI and NgoMIV restriction sites. To generate the RCASBP(A)-*miR* vector, a recombination between the pENTR3C-*miR* entry vector and RCASBP(A)-YDV gateway destination vector was performed using a LR clonase kit (Invitrogen).

### Dual luciferase reporter assay

DF1 cells were infected with either RCAS-*ssc-miR-147*, RCAS-*ssc-miR-24*, RCAS-*ssc-miR146a* or RCAS-*SC* (M.O.I. of 1) and maintained in a 96-well plate in RPMI 1640 with 1% heat-inactivated FBS, L-glutamine, penicillin (100 U/ml), streptomycin (100 µg/ml), and fungizone (4 µg/ml), at 37°C with 5% CO_2_. At 3 dpi, each psiCHECK-2 target construct (100 ng) was transfected (in triplicate) into the DF1 cells using FuGENE 6 (Promega). Forty-eight hours post-transfection, cells were lysed in Passive Lysis Buffer (Promega), firefly and *Renilla* luciferase activities were then assessed using the Dual-Luciferase Reporter Assay System (Promega) and a VictorLight 1420 luminescence counter (PerkinElmer). Normalized luciferase activity was calculated from the *Renilla/*firefly signal ratio. A Student's t-test (p<0.05) was used to determine repression of the *Renilla* reporter gene by a given miRNA by comparing the relative luciferase activity between cells infected with an RCAS expressing the miRNA and the RCAS expressed the scrambled control sequence. The assay was independently repeated to confirm the results.

### Over-expression of *miR-147* using a synthetic mimic

Primary alveolar macrophages from two six-week old pigs were seeded at a density of 1.3×10^6^ cells per well. All transfections and infections were performed in duplicate. Cells were transfected with either 100 nmol of a *miR-147* mimic or a negative control mimic (Dharmacon) using siPORT amine (Life Technologies) according to the manufacturer's instructions. At 16 hours post transfection macrophages were infected with PRRSV (VR-2332) at M.O.I. 1. At 8 hpi, 12 hpi, and 24 hpi total RNA was isolated using Qiazol (Qiagen) and overnight precipitation at -20°C. RNA was treated with DNase using a TURBO DNase kit (Life Technologies) following the manufacturer's instructions. For miRNA expression analysis 1 µg of total RNA was reversed transcribed using a miRNA 1^st^-strand cDNA synthesis kit (Agilent Technologies) following the manufacturer's instructions. For gene expression analysis 1 µg of total RNA was reverse transcribed using a SuperScript III kit (Life Technologies). All Real-Time PCR reactions were performed in duplicate. For small RNA expression analysis each reaction contained 10 ng of cDNA, 500 nmol of gene-specific forward primer, 312 nmol Universal Reverse primer (Agilent) and 1X iQ SYBR Green Supermix (Bio-Rad). The following PCR conditions were used: 95°C for 15 minutes, followed by 40 cycles of 95°C for 10 seconds, then 58°C for 20 seconds. Gene specific amplification was confirmed using melting curve analysis. The same conditions were also used for gene expression analysis with the exception that 500 nmol of both a gene-specific forward primer and a gene-specific reverse primer was used (Table S1). Threshold cycle (Ct) values were transformed to a relative expression in arbitrary units by the 2^−ΔΔCt^ method [10] and normalized to expression levels of *sno83b* or *RPL4* for small RNA and gene expression analysis, respectively. TCID_50_ virus titers were determined via immunofluorescence staining of the PRRSV nucleocapsid protein, using monoclonal antibody SDOW17 (Rural Technologies, INC) and calculated by the Reed-Muench method.

## Results

### Characteristics of the small RNA libraries

To investigate the miRNAome of PAMs and the effect of PRRSV infection on miRNA expression small RNA libraries from PRRSV-infected PAMs *in vitro* were analyzed using Illumina deep sequencing. The total number of reads obtained for each library ranged from 704,925 to 1,107, 968. The total number of high quality reads (Phred ≥20) ranged from 499,848 to 790,003. Of the total 13,204,508 high quality reads obtained, 12,626,941 (95.6%) aligned with the *Sus scrofa* genome. The total number of known porcine miRNAs identified in each library ranged from 104 to 124 and the number of homologous miRNAs per library ranged from 187 to 262. The ten most commonly sequenced miRNAs in swine alveolar macrophages are listed in Table 1. The highest expressed miRNA was *ssc-miR-21* which represented ∼37% of the total miRNA reads.

**Table 1:** Most frequently sequenced miRNAs in swine alveolar macrophages^a^.

miRNA	Average CPM^b^	% of total miRNA^c^
*ssc-miR-21*	366,222	36.66
*ssc-miR-92a*	80,385	8.05
*ssc-miR-146b*	70,906	7.10
*ssc-let-7i*	34,285	3.43
*ssc-let-7f*	26,580	2.66
*ssc-miR-191*	25,096	2.51
*ssc-miR-30d*	19,691	1.97
*ssc-miR-532-5p*	19,193	1.92
*ssc-miR-23a*	16,474	1.65
*ssc-miR-27a*	16,217	1.62

a Small RNA libraries were generated from alveolar macrophages from three 7-week old pigs and cultured for 24 hours.

b Values are given as average counts per million (cpm) reads.

c percentage of total normalized cpm reads matching known and homologous miRNAs.

### Differentially expressed miRNAs in PRRSV infected PAMs

**Figure 1:**
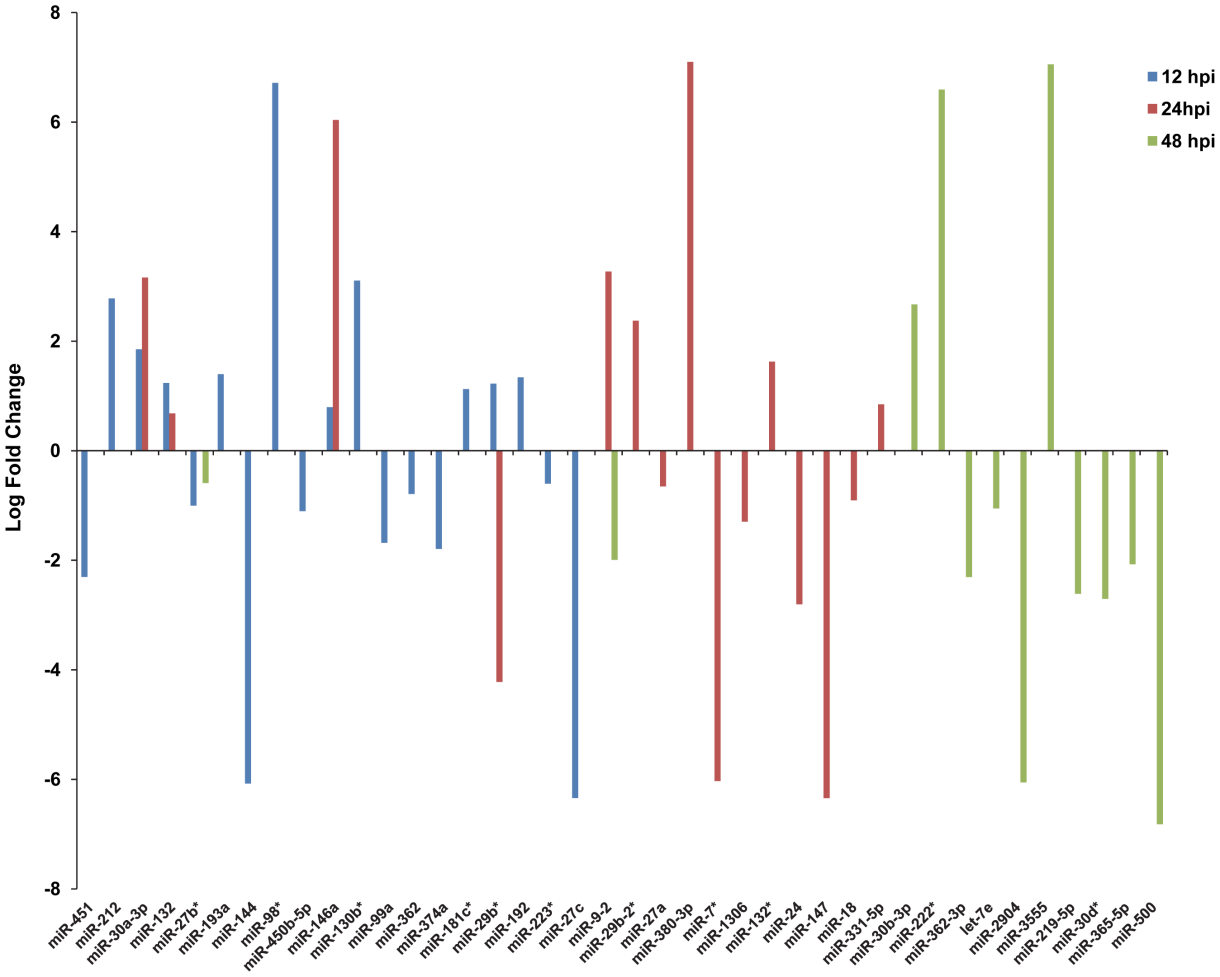
Differentially expressed cellular miRNAs in PRRSV-infected PAMs. PAMs from three 7-week old pigs were infected with PRRSV (strain VR-2332) at M.O.I. 10 and small RNA expression was determined at 12 hpi, 24 hpi, and 48 hpi using Illumina deep sequencing. A total of forty cellular miRNA were significantly differentially expressed within the first 48 hpi (p<0.05) and are listed along the x-axis. The log fold expression differences of these miRNAs are shown on the y-axis. At 12 hpi (blue bars) 19 miRNAs were differentially expressed, at 24 hpi (red bars) 15 miRNAs were differentially expressed and at 48 hpi (green bars) 12 miRNAs were differentially expressed. Among these six miRNA, *miR-30a-3p*, *miR-132*, miR-27b*, *miR-29b*, *miR-146a* and *miR-9-2*, were altered at more than one time point.

In order to determine what effect, if any, that PRRSV infection has on the porcine alveolar macrophage miRNAome, pairwise comparisons were performed between time-matched mock and VR-2332 infected PAMs. Overall, the expression of 40 miRNAs was significantly (p<0.05) altered in PRRSV-infected PAMs at one or more time points compared to mock-infected controls (Figure 1). The expression of 19 miRNAs was significantly altered in PRRSV-infected PAMs at 12 hpi. Of the 19 differentially expressed miRNAs at 12 hpi the miRNAs displaying the highest log fold changes were miR-98* (∼6.7) and *miR-27c* (∼−6.3). At 24 hpi 15 miRNAs were found to be differentially expressed between PRRSV-infected and mock-infected PAMs. At 24 hpi, the miRNAs with the largest log fold changes in expression were *miR-380-3p* (∼7.1) and *miR-147* (∼−6.3). Twelve miRNAs displayed altered expression patterns in PRRSV-infected PAMs at 48 hpi. The most differentially expressed miRNA at 48 hpi in PRRSV-infected PAMs was *miR-3555* which had ∼7 log fold increase in PRRSV-infected PAMs compared to mock-infected macrophages (Figure 1). The next most altered miRNA was *miR-500* which had a log fold change of ∼−6.8 in PRRSV-infected PAMs. Of the differentially expressed miRNAs six (*miR-30a-3p*, *miR-132*, miR-27b*, *miR-29b*, *miR-146a* and *miR-9-2*) were altered at more than one time point in PRRSV-infected macrophages (Figure 1).

### Target prediction analysis and validation of differentially expressed miRNAs

**Figure 2:**
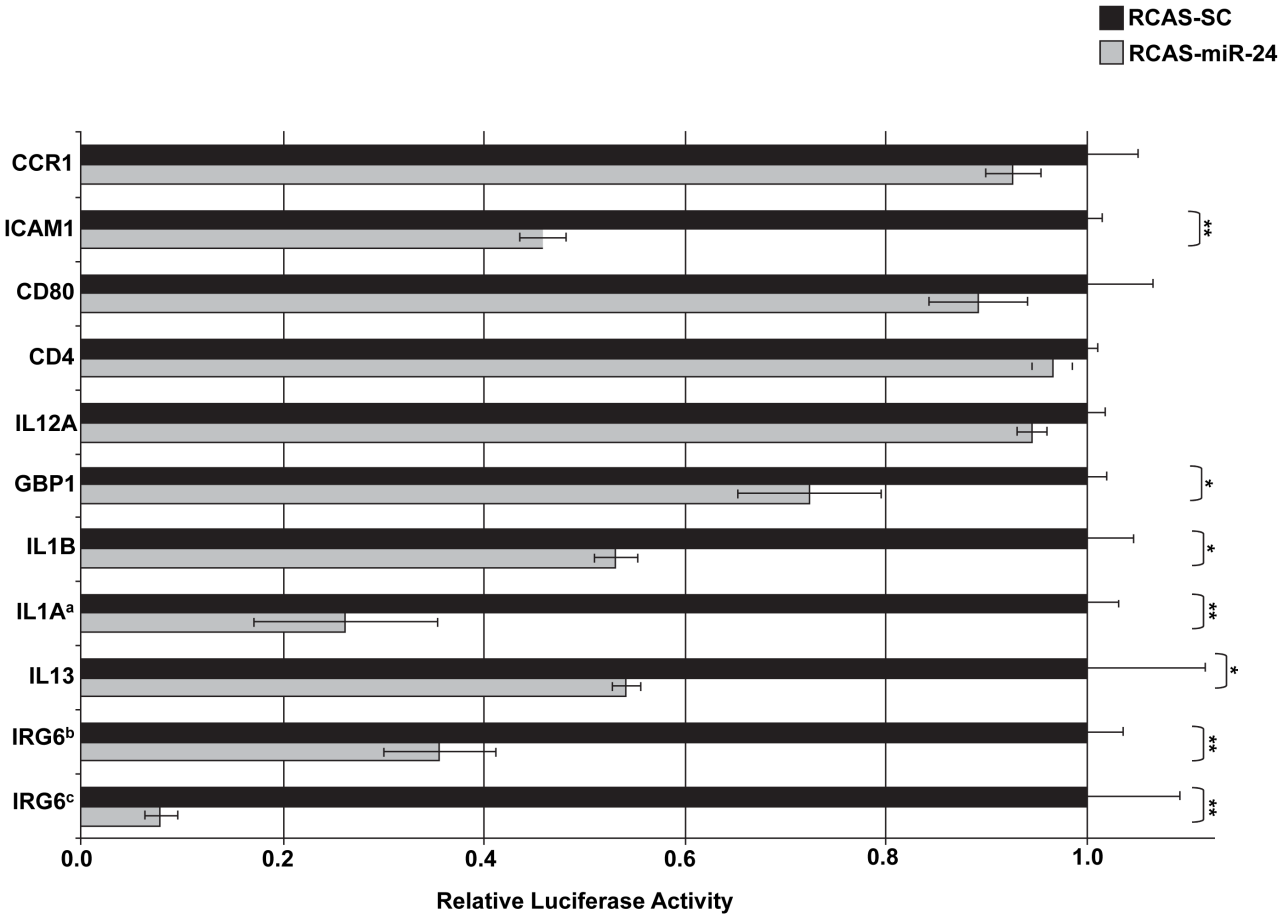
Validation of predicted *ssc-miR-24* target genes. To confirm the legitimacy of selected miR-24 binding sites, a previously developed [REF] in vitro luciferase assay was utilized. Gateway cloning (Life Technologies) was used to create RCAS viruses expressing either the *ssc-miR-24* hairpin or a scrambled control (*SC*) hairpin. DF1 cells, a chicken embryonic fibroblast cell line, were infected with either the RCAS-*miR-24* expressing virus or the RCAS-*SC* expressing virus were transfected with predicted target 3′ UTR luciferase constructs. Target constructs were generated by cloning the miRNA binding site and its flanking regions downstream of the Renilla luciferase gene in the psiCHECK2 vector (Promega). Relative *Renilla* luciferase activity is shown. *: p<0.05, **: p<0.01. ^a^This construct contains two *miR-24* binding sites. ^b^This construct contains two *miR-24* binding sites. ^c^This construct contains three *miR-24* binding sites.

**Figure 3:**
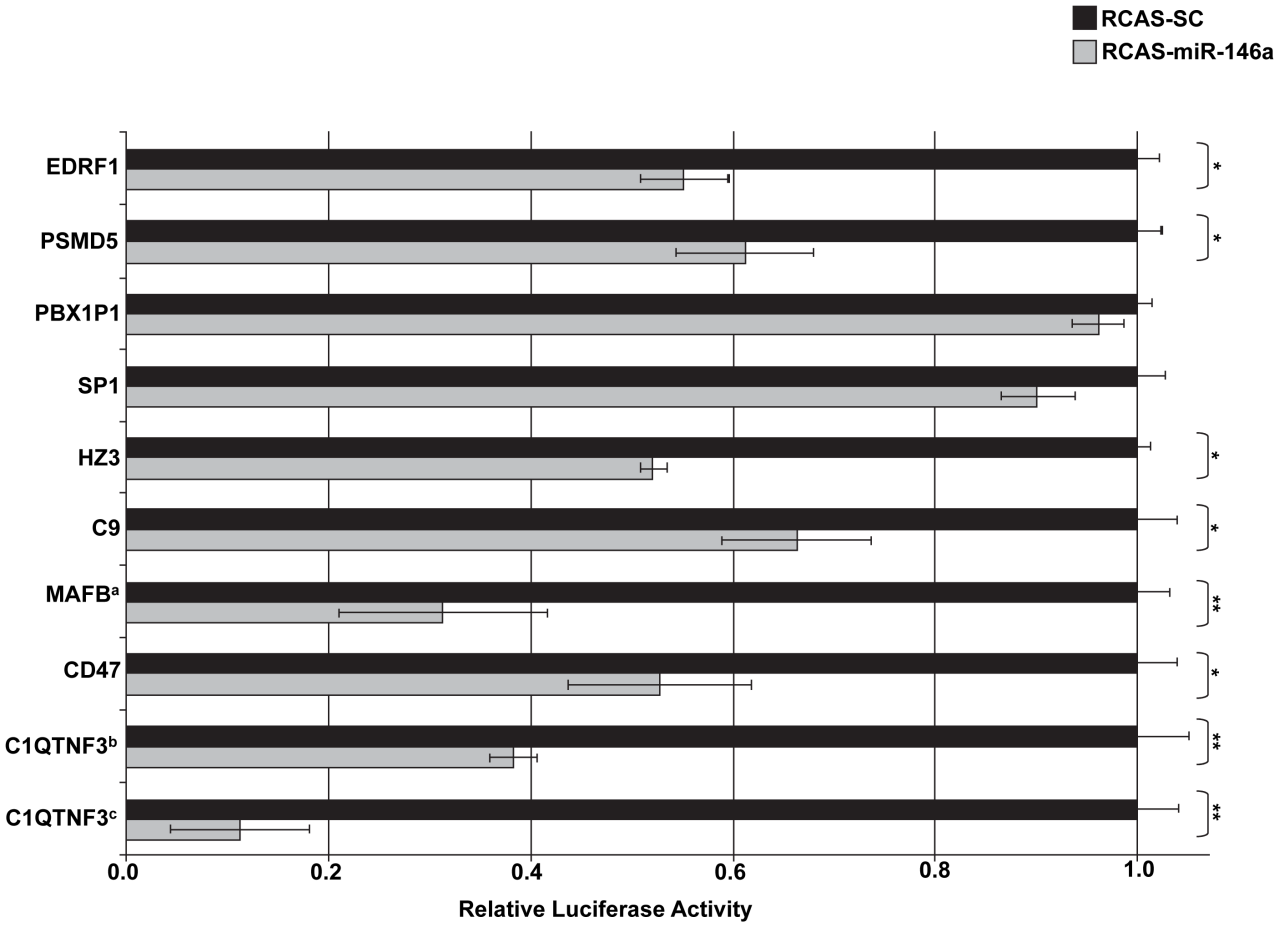
Validation of predicted *ssc-miR-146a* target genes. To validate predicted porcine binding sites for *miR-146a* an RCAS virus expressing *the ssc-miR-146a* hairpin was generated using the Gateway system (Life Technologies). RCAS-infected DF1 cells expressing either *miR-146a* or a scrambled control (SC) were transfected with psiCHECK2 constructs in which the predicted *miR-146a* binding sites along with their flanking regions were cloned into the 3′UTR of the Renllia luciferase gene. Relative *Renilla* luciferase activity is shown. *: p<0.05, **: p<0.01. ^a^This construct contains two *miR-146a* binding sites. ^b^This construct contains one *miR-146a* binding site. ^c^This construct contains two *miR-146a* binding sites.

**Figure 4:**
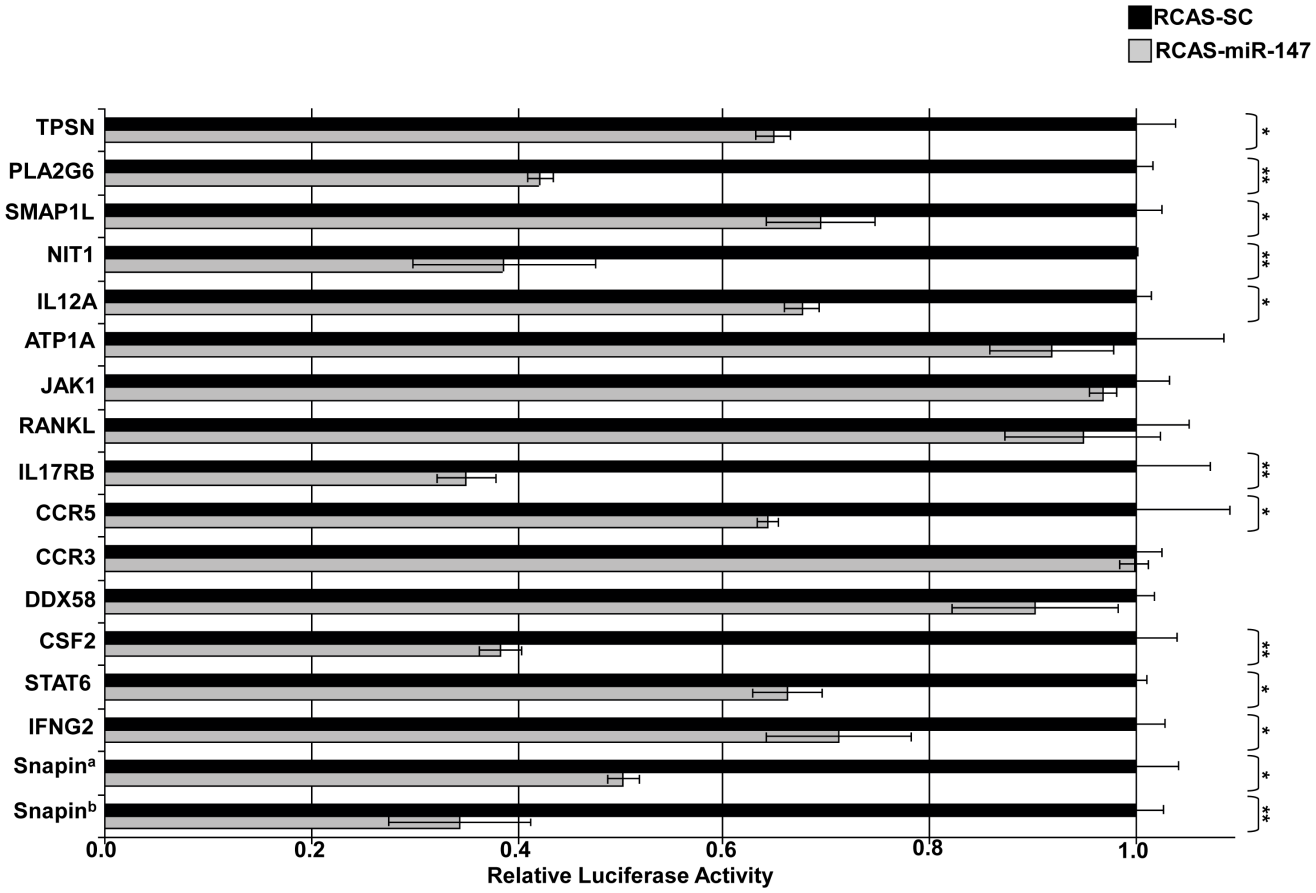
Validation of predicted *ssc-miR-147* target genes. An RCAS-based miRNA expression was utilized to authenticate selected predicted *miR-147* target sites. The hairpin for *ssc-miR-147* was cloned into RCASBP(A)-YDV using Gateway cloning (Life Technologies). DF1cells ectopically expressing *miR-147* or a scrambled control (SC) were transfected with psiCHECK2 constructs containing *miR-147* binding sites cloned downstream of the *Renilla* luciferase gene. Relative *Renilla* luciferase activity is shown. *: p<0.05, **: p<0.01. ^a^This construct contains one *miR-147* binding site. ^b^This construct contains two *miR-147* binding sites.

Three miRNAs, *miR-24*, *miR-146a*, and *miR-147* were selected for target gene validation based on their expression profiles, predicted regulated pathways and/or their known involvement in immunity. Target prediction and subsequent validation of selected putative targets for these miRNAs via a luciferase assay suggest that they likely regulate numerous cellular pathways including immune and intracellular trafficking pathways. Of the ten potential *miR-24* regulated genes selected for binding site validation, the *miR-24* binding sites of six genes (*IRG6*, *IL1A*, *IL1B*, *IL13*, *GBP1*, and *ICAM1*) significantly reduced *Renilla* luciferase expression in DF1 cells infected with RCAS-*ssc-miR-24* compared to cells infected with RCAS-*SC* (Figure 2). The 3′UTR of *IRG6* has three potential *miR-24* binding sites (Table 2). When two of these binding sites were inserted into the 3′UTR of *Renilla* luciferase, its expression was knocked-down ∼50%, when all three sites were included *Renilla* expression was further repressed (Figure 2). Seven of the predicted *ssc-miR-146a* regulated genes (*C1QTNF3, C9, CD47, MAFB, HZ3, PSMD5*, and *EDRF1*) out of the nine selected for validation were able to significantly reduce *Renilla* luciferase expression (Figure 3). Both *C1QTNF3* and *MAFB* contain two putative *miR-146a* binding sites within their 3′UTRs (Table 3). For *ssc-miR-147* sixteen potential regulated genes were selected for validation using the luciferase assay. The *miR-147* binding sites for eleven of these genes (*Snapin, IFNGR2, STAT6, CSF2, CCR5, IL17RB, IL12A, NIT1, SMAP1L, PLA2G6*, and *TPSN*) were successful in significantly reducing *Renilla* luciferase expression (Figure 4). The 3′UTR of *Snapin* was predicted to contain 2 *miR-147* binding sites (Table 4). When both sites were included in the *Renilla* luciferase construct, there was a greater repression of luciferase activity than when only a single site was included (Figure 4).

**Table 2:** Computationally predicted porcine *miR-24* target sites chosen for *in vitro* validation.

Symbol/GI	mRNA binding site^a^	Score/Energy (kcal/mol)	Location
**C1QTNF3/194719516**	**5′ ttCCCA--AAAATCAGTTCTCA 3′**	**169/-21.7**	**2325–2344**
	**5′ tcCTCATGGATACTAGTTGGGTTCTTA 3′**	**141/-21.2**	**2015–2041**
**CD47/47522789**	**5′ tGCCCAATTGAGATCCAGTTCTTt 3**	**155/-18.9**	**1257–1280**
**MAFB/194033944**	**5′ AAAGCGTATTTTTTAGTTCTCA 3′**	**159/-17.8**	**2607–2628**
	**5′ ctCCCGT----TTTAGTTTTCA 3**	**147/-18.9**	**2015–2032**
**C9/86604372**	**5′ ctCCTATGGTTTCCAGTTTTTt 3′**	**140/-20.4**	**1960–1981**
**HZ3/194043551**	**5′ AACCC-TGTGAA---AGTTCTCA 3′**	**167/-20.8**	**3433–3451**
**SP1/194037363**	**5′ tACCAGTGGCAAGGCAGTTCTTt 3′**	**153/-20.8**	**3804–3826**
**PBXIP1/194036092**	**5′ ccCCCTTTTGGCCCAGTTCTTc 3′**	**136/-18**	**2299–2320**
**PSMD5/194033944**	**5′ GAACCA--GA---CAGTTCTCA 3′**	**156/-17.2**	**1443–1459**
**EDRF1/194041642**	**5′ GAGCCA--GGGTGCAGTTCTCt 3′**	**162/-22.6**	**1866–1885**

a Capitalized text indicates basing pairings with the microRNA.

**Table 3:** Computationally predicted porcine *miR-146a* target sites selected for validation.

Symbol/GI	mRNA binding site^a^	Score/Energy (kcal/mol)	Location
**IL1A/1987**	**5′ aTGCTTCT--TGAAGCTGAGCCt 3′**	**165/-23.1**	**1493–1513**
	**5′ taGTCACTCGCTGAGCATGTGCTGAGCCt 3′**	**154/-22.8**	**931–959**
**IL1B/47522925**	**5′ cccaTCCTCAGGCCCCATCCACTGAGCCA 3′**	**153/-21**	**1061–1089**
**IL13/189176150**	**5′ gatTTCCTTAGCTTAGACTTGAGCCt 3′**	**151/-18.9**	**581–606**
**IRG6/47523213**	**5′ ggGGTGCTGTGTGAATCTCCTGAGTCt 3′**	**142/-18.1**	**1737–1763**
	**5′ aTG-TCAT-TTGAACTGGGTCA 3′**	**140/-22.2**	**2058–2077**
	**5′ CTGATGAGTCTGAAGCCCAAATCCTGAGTCA 3′**	**135/-17.6**	**2683–2713**
**GBP1/166202345**	**5′ CTG----TGCTTCACTGAGCTt 3′**	**144/-17.8**	**2956–2973**
**IL12A/47522811**	**5′ gTGTT--TGTAGAAACAAACACTTGAGCCt 3′**	**136/-20.1**	**1248–1275**
**CD4/47641126**	**5′ CTG----TGCTTCACTGAGCTt 3′**	**138/-18.6**	**2652–2673**
**CD80/55742787**	**5′ CTCTCACAGTAGTGAGAATTCTGAGCCc 3′**	**146/-19.7**	**1606–1636**
**ICAM1/55742637**	**5′ CTGTAGCCACAGCACTGAGCCA 3′**	**167/-21.7**	**1855–1876**
**CCR1/44890871**	**5′ ggcTTCTAGTTGTACGGCTGAGCgt 3′**	**139/-16.8**	**2398–2422**

a Capitalized text indicates basing pairings with the microRNA.

**Table 4:** Computationally predicted porcine *miR-147* target sites chosen for experimental validation.

Symbol/GI	mRNA binding site^a^	Score/Energy (kcal/mol)	Binding site
**SNAPIN/194036138**	**5′ GCTGAGGC--TTCTACACAT 3′**	**153/-19.8**	**649–666**
	**5′ GGAGAGGCTTCCTCTGCTACACGC 3′**	**131/-19.3**	**733–756**
**IFNGR2/162287024**	**5′ tgtttAGCATGATTCTACATAC 3′**	**140/-17.6**	**1513–1534**
**STAT6/194037565**	**5′ agGGGGGCA---CCACGCAC 3′**	**140/-21.2**	**4367–4383**
**CSF2/47523043**	**5′ cCAGAGTCCCACCTTCCACACAg 3**	**157/-19.7**	**487–510**
**DDX58/47523231**	**5′ tctaAAGC---TTCACACAC 3′**	**142/-16.2**	**3450–3466**
**CCR3/44890873**	**5′ GTGGAGGTATATCTATGCAC 3′**	**133/-18.9**	**1588–1607**
**CCR5/44890879**	**5′ cttgcctcccTTCCACATAC 3′**	**149/16.1**	**1326–1345**
**IL17RB/194041250**	**5′ GCAGGTGAAAATCCATATAT 3′**	**133/-16.7**	**1696–1715**
	**5′ aaGGAAGGA--TTCATACAC 3′**	**131/-16.9**	**1869–1886**
**RANKL/194040708**	**5′ aTGGAAGGGTTTTCTTACACAg 3′**	**133/-18.7**	**1140–1161**
**JAK1/6635249**	**5′ GCTGAATGCATATATACCACGTAC 3′**	**137/-18.4**	**4585–4608**
**ATP1A1/47523569**	**5′ cgtGGAGCATCACGCCACATAC 3′**	**144/-22.1**	**3116–3137**
**IL12A/47522811**	**5′ tgtGAGGC-TCCCATCCACACAa 3′**	**148/-19**	**1053–1074**
**NIT1/194036899**	**5′ cCAGAGTCCCACCTTCCACACAg 3′**	**157/-19.6**	**2044–2066**
**SMAP1L/183223972**	**5′ tCAAAGG---TCCCACACAC 3′**	**153/-16.6**	**2385–2401**
**PLA2G6/194037216**	**5′ cCAGCAGCTCTGTGTCCACACAC 3′**	**168/-23.3**	**2475–2497**
**TPSN/194040292**	**5′ cCACAGCCACAGCCACACAg 3′**	**151/-17.1**	**1652–1671**

a Capitalized text indicates basing pairings with the microRNA.

### Over expression of *miR-147* using a synthetic mimic

**Figure 5:**
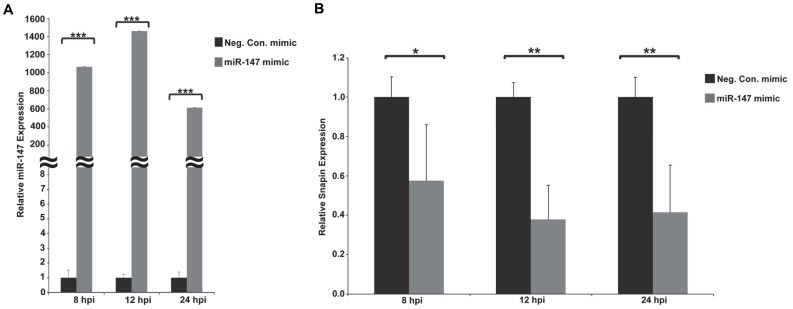
Over expression of *miR-147* in PRRSV infected PAMs using a synthetic mimic. PAMs were transfected with 100*miR-147* mimic (Dharmacon) or a negative control mimic (Dharmacon) using amine-based transfection. Sixteen hours post-transfection, PAMs were infected with PRRSV (VR-2332) at M.O.I. 1. A. Real-time RT-PCR analysis of *miR-147* expression in PAMs transfected with either the *miR-147* mimic (grey bars) or the negative control mimic (black bars). ***p≤0.001. B. Real-time RT-PCR analysis of *Snapin*, a *miR-147* target, in PRRSV-infected PAMs transfected with a *miR-147* mimic (grey bars) or a negative control mimic (black bars). *p≤0.05, **p≤0.01.

**Figure 6:**
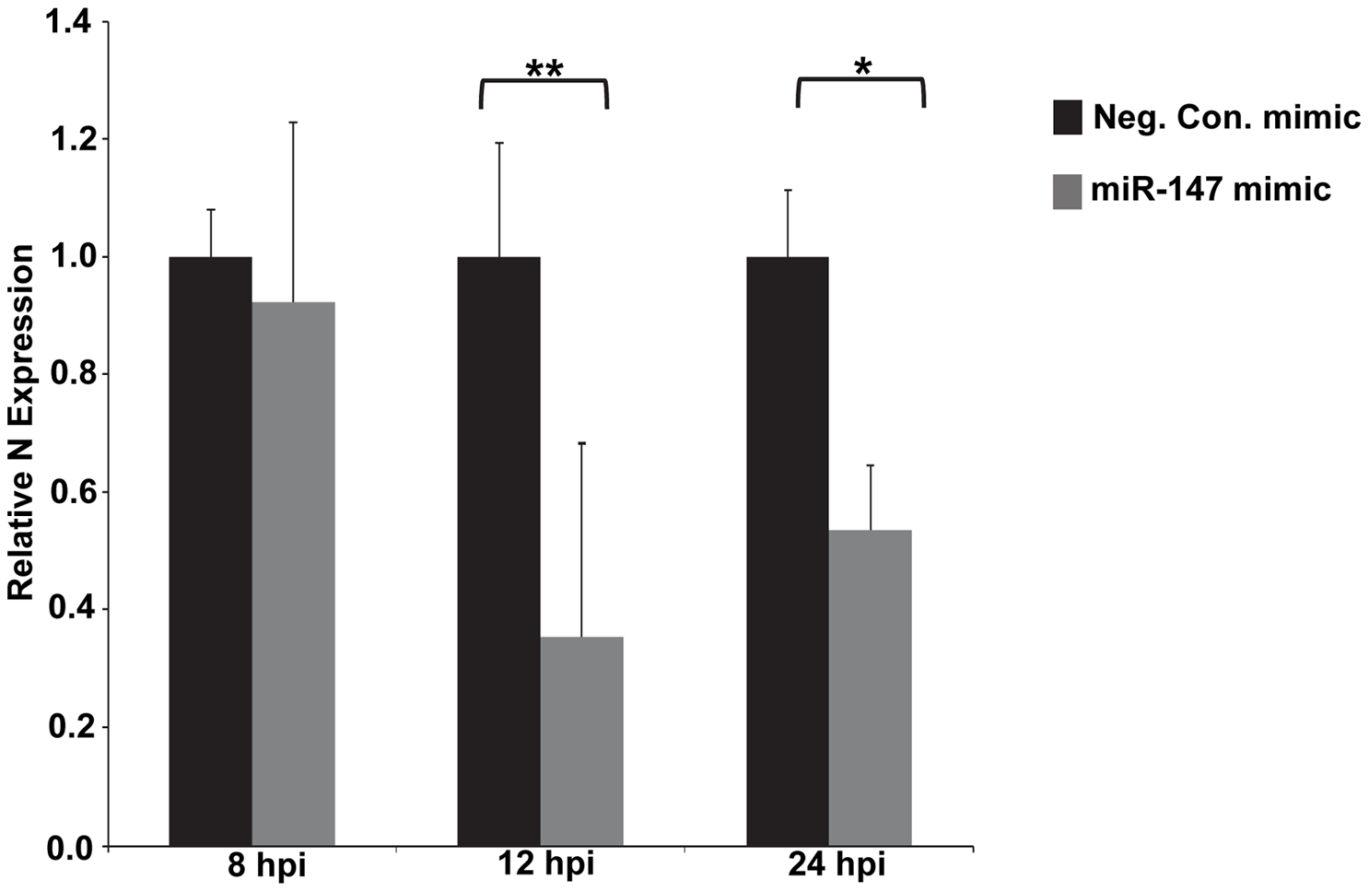
PRRSV *N* levels in PAMs transfected with either a *miR-147* mimic or a negative control mimic. PAMs were transfected with 100*miR-147* mimic (Dharmacon) or a negative control mimic (Dharmacon) using amine-based transfection. Sixteen hours post-transfection PAMs were infected with PRRSV (VR-2332) at M.O.I. 1. Total RNA was isolated at 8 hpi, 12 hpi and 24 hpi. Real-time RT-PCR analysis of the PRRSV nucleocapsid protein mRNA levels in PAMs transfected with either a *miR-147* mimic (grey bars) or a negative control mimic (black bars). *p≤0.05, **p≤0.01.

**Figure 7:**
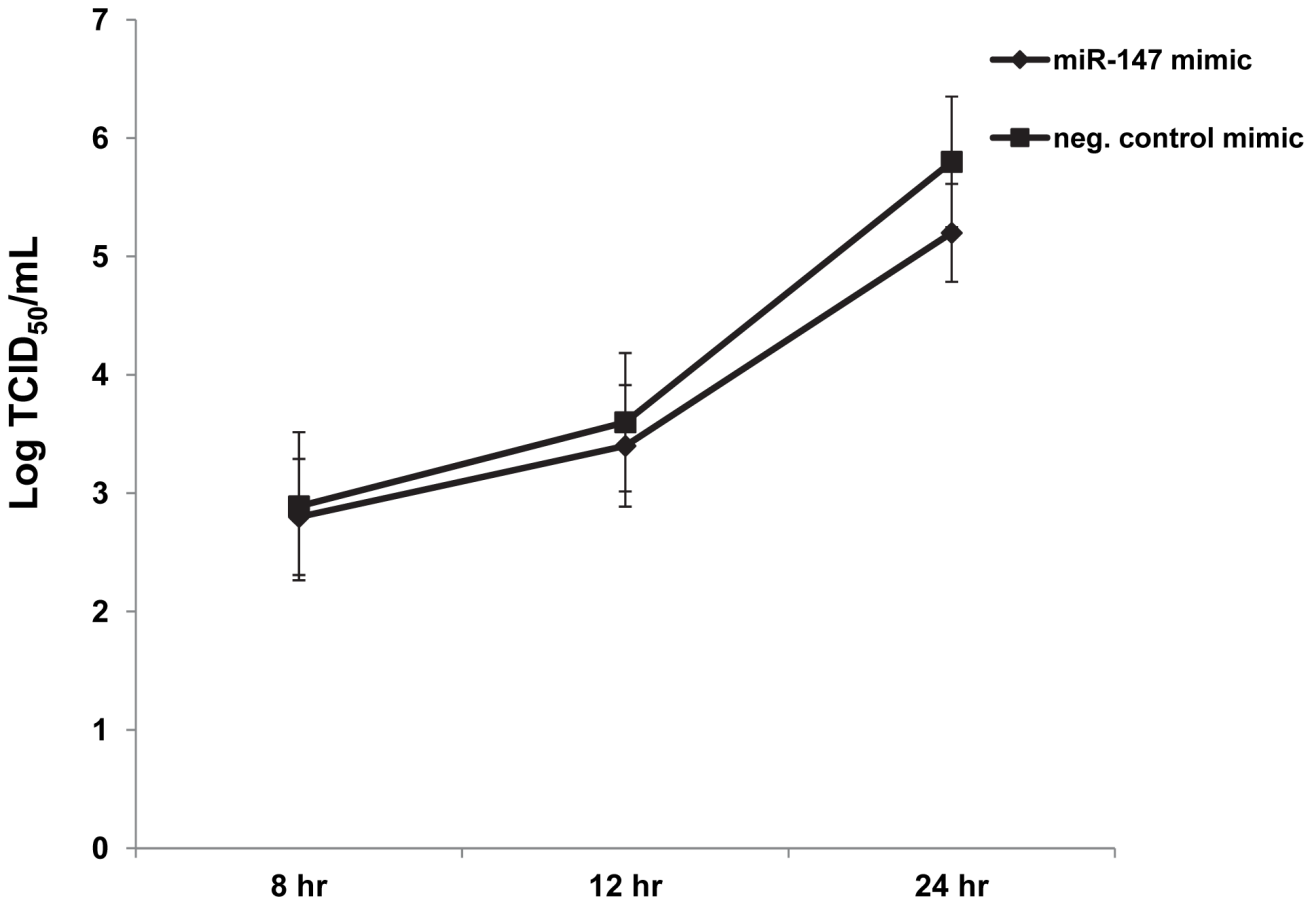
PRRSV titers in PAMs transfected with either a miR-147 mimic or a negative control mimic. PAMs were transfected with 100-147 mimic (Dharmacon) or a negative control mimic (Dharmacon). PAMs were infected with PRRSV (VR-2332) at M.O.I. 1. Supernatants were collected at 8 hpi, 12 hpi, and 24 hpi. TCID_50_ viral titers were determined using the Reed- Muench method and immunofluorescence staining of the PRRSV nucleocapsid protein. Assay was repeated to confirm results.

The cellular miRNA *miR-147* was the most repressed miRNA 24 hours post PRRSV infection of alveolar macrophages, with a log fold change of −6.3. To investigate potential consequences of this down-regulation in PRRSV-infected cells, a synthetic *miR-147* mimic was used to maintain high *miR-147* levels during infection. Transfection of the *miR-147* mimic increased *miR-147* levels in PAMs several hundred fold compared to the negative control mimic (Figure 5A). To confirm the increased *miR-147* functionality in transfected *miR-147* transfected PAMs, real-time PCR analysis of the *miR-147* target gene *Snapin*, which contains two *miR-147* binding sites within its 3′UTR, was performed (Figure 5B). There was a significant decrease in *Snapin* levels in PAMs transfected with the *miR-147* mimic compared to the negative control mimic (Figure 5B) verifying increased *miR-147* targeting. PAMs therefore appear to be amiable amine-based transfection. To determine the potential impact of *miR-147* gene regulation on PRRSV real-time PRRSV analysis of the PRRSV nucleocapsid was performed. PRRSV N protein mRNA levels did not significantly differ between *miR-147* mimic-transfected PAMs and negative control mimic-transfected cells at 8 hpi (Figure 6). However, at both 12 hpi and 24 hpi PRRSV N protein mRNA levels were significantly reduced in *miR-147* mimic-transfected PAMs (Figure 6). To further assess the potential influence of *miR-147* over-expression on PRRSV, viral titers were also determined. There was a small but perceptible reduction in PRRSV production in *miR-147* mimic-transfected PAMs compared to negative control mimic-transfected PAMs (Figure 7).

## Discussion

It has been well established that cellular miRNAs are important regulators of animal immunity and viral pathogenesis. The present study was undertaken to determine the changes in cellular miRNA expression in porcine alveolar macrophages in response to PRRSV infection *in vitro*. To this end, small RNA profiles of PRRSV infected PAMs at 12 hpi, 24 hpi, and 48 hpi were generated using Illumina deep sequencing. Approximately 120 known porcine miRNAs and ∼200 homologous miRNAs were found to be expressed in PAMs. This suggests that miRNAs are likely important regulators in porcine macrophages. A total of forty cellular miRNAs were differentially expressed in PRRSV infected macrophages, six of which (*miR-30a-3p*, *miR-132*, miR-27b*, *miR-29b*, *miR-146a* and *miR-9-2*) were altered at more than one time point (Figure 1). To investigate potential functions of these miRNAs, *in silico* target prediction analysis and subsequent *in vitro* validation was performed. Moreover, functional characterization of one of these differentially expressed miRNAs, *miR-147*, suggests cellular miRNAs may have an effect on PRRSV replication.

The expression of the miRNA *miR-24* was significantly down-regulated in PRRSV infected PAMs at 24 hpi (Figure 1). Target prediction analysis suggests that *miR-24* is involved in the regulation of several immune-associated genes (Table 2, Figure 2). Porcine inflammatory response protein 6 (IRG6; also known as RSAD2 or viperin) contains three potential *miR-24* binding sites in its 3′UTR (Table 2). When cloned into the 3′UTR of *Renilla* luciferase these *miR-24* binding sites were able to significantly reduce *Renilla* expression in DF1 cells over-expressing porcine *miR-24* (Figure 2). IRG6 is known to function as an anti-viral protein and is induced upon viral infections or LPS simulation in many different cell types (reviewed by [11]). Typically IRG6 levels are low in resting cells and are induced upon immune stimulation [11]. As *miR-24* was found to be significantly down-regulated in PRRSV-infected PAMs compared to mock-infected cells it would be expected that IRG6 levels should be induced in PRRSV infected cells. One possible function of *miR-24* is to maintain reduced levels of IRG6 in healthy cells and then upon viral infection *miR-24* expression is down-regulated resulting in increased IRG6 expression and subsequent induction of an anti-viral response. Two related cytokines, interleukin one alpha (IL1A) and interleukin one beta (IL1B), have potential *miR-24* binding sites in their 3′UTRs (Table 2). Treatment of LPS-stimulated RAW-264.7 cells with melatonin results in increased expression of both IL1A and IL1B [12], indicating a possible role for these cytokines in the macrophage inflammatory response. A recent meta-analysis of both the general porcine immune response as well as the PRRSV-specific response identified over 500 genes with a PRRSV-specific response [13]. Among these genes are IL1A and ILB. Therefore one possible explanation for the increased expression of IL1 during PRRSV infection [14] may be, in part, the down-regulation of *miR-24*. IL1A and IL1B are pro-inflammatory cytokines which are often co-induced and function in the activation of the NFκB signaling pathway [15]. NFκB activation is induced upon PRRSV infection and is a key component of PRRSV pathogenesis [16]. The results of the present study add to the already known complex regulation of NFκB expression during PRRSV infections by suggesting that this activation, in part, may be due to decreased expression of *miR-24* in PRRSV- infected cells and thus results in an increase in expression of IL1A and IL1B. The chemokine (C-C motif) receptor (CCR1) was also found to be part of the PRRSV-specific response [13]. CCR1 also contains a *miR-24* binding site (Table 2). When the CCR1 *miR-24* binding site was inserted into the 3′UTR of Renilla luciferase there was a slight decrease in luciferase activity, suggesting that *miR-24* may regulate CCR1 expression during the response to PRRSV infection. However this needs to be further experimentally validated.

In an effort to identify genes associated with the immune response to PRRSV infection, gene expression differences between pigs presenting with a high PRRSV viral load and pigs presenting with a low PRRSV viral load were identified [17]. Among the genes up-regulated in high viral load pigs is feline leukemia virus subgroup C receptor-related protein 2 (FLVCR2). *In silico* target prediction for miRNAs differentially expressed in PRRSV-infected PAMs, identified two potential *miR-24* binding sites within the 3′ UTR of FLVCR2. FLVCR2 functions as a calcium transporter [18]. Intracellular calcium transport and metabolism has been shown to be involved in PRRSV infections. Multiple genes involved in intracellular calcium homeostasis are differentially expressed in the lungs of pigs infected with highly pathogenic PRRSV [19]. Piglets congenitally infected with PRRSV display increased levels of toll-like receptor three (TLR3) [20]. Furthermore, over-expression of TLR3 results in an increased influx of calcium upon immune stimulation. Therefore, one of the possible functions of *miR-24* down-regulation during PRRSV infections may be to increase the expression of FLVCR2 and subsequently modulate intracellular calcium levels.

The miRNA *miR-146a* was up-regulated at both 12 hpi and 24 hpi infection in PRRSV-infected macrophages. Hyaluronan synthase 3 (HAS3), a regulator of hyaluronan synthesis, possesses two potential binding sites for *miR-146a* in its 3′ UTR (Table 3). HAS3 is down-regulated in PRRSV-infected pigs displaying a high viral load compared to pigs with low viral loads [17]. Thus it is possible that this down-regulation of HAS3 is, in part, due to the up-regulation of *miR-146a* in PRRSV-infected cells. A related hyaluronan synthase, HAS1, was recently linked to M. tuberculosis infections of macrophages [21]. It was demonstrated that a HAS1-specific siRNA inhibitor reduced macrophage susceptibility to M. tuberculosis infection. Therefore, the potential targeting of HAS3 by *miR-146a* may impact PRRSV infectivity.

*MiR-146a* is one of the better characterized vertebrate miRNAs and is known to play various roles in the immune system (reviewed by [22]). The expression of *miR-146a* is induced in monocytes and other immune cells upon exposure to pathogens. This up-regulation in expression is thought to be dependent upon NFκB activation. *MiR-146a* has previously been shown to regulate a diverse group of genes including immune response modulators such as, TRAF6, IRAK1, IRAK2, IL-8 and RANTES (reviewed by [22]). In the present study *miR-146a* was differentially expressed at both 12 hpi and 24 hpi and displayed dramatic up-regulation at 24 hpi in PRRSV-infected PAMs (Figure 1). Binding site prediction and validation identified additional genes regulated by *miR-146a*, including *C1QTNF3* and *MAFB* (Table 3 and Figure 3). Tumor necrosis factor related protein 3 (C1QTNF3) is predicted to contain two *miR-146a* binding sites in its 3′UTR. C1QTNF3 is a recently identified cytokine involved in the anti-inflammatory response of monocytic cells [23]. As *miR-146a* expression is significantly higher in PRRSV infected PAMs compared to mock infected cells, C1QTNF3 expression would therefore be expected to be lower in PRRSV infected cells. This potential decrease of C1QTNF3 may serve to aid in the immune response to PRRSV infection. MAFB, v-maf musculoaponeurotic fibrosarcoma oncogene homolog B (avian), is also predicted to contain two potential *miR-146a* binding sites within its 3′UTR (Table 3) and luciferase validation suggests that these sites are functional (Figure 3). MAFB is a transcription factor which, among other roles, functions in macrophage differentiation [24]. MAFB was recently shown to be involved in the response of murine macrophages stimulated with either TLR agonists or subjected to *Salmonella* infection [25]. This suggests that MAFB-mediated gene regulation is likely associated with the response of macrophages to pathogenic infections. MAFB expression was recently associated with both the response to PRRSV infections as well as the general immune response in pigs [13]. Based on the roles of *miR-146a* in the regulation of immune responsive genes and its up-regulation in PRRSV-infected PAMs, it is likely that *miR-146a* mediated gene regulation is an important aspect of the host response to a PRRSV infection.

The miRNA *miR-147* had a log fold difference of ∼−6.3 in PRRSV infected PAMs compared to mock infected PAMs at 24 hpi (Figure 1). Among the potential porcine genes predicted to contain *miR-147* binding sites was *Snapin* (Table 4). Snapin contains two potential *miR-147* binding sites within its 3′UTR and the results of the luciferase validation assay suggest that these sites are functional (Figure 4). Other potential *miR-147* regulated genes include *CSF2* (Table 4 and Figure 4). Colony stimulating factor 2 (granulocyte-macrophage) or CSF2 is a cytokine that controls the production, differentiation, and function of granulocytes and macrophages [26]. CSF2 was recently shown to function in the antiviral response to influenza A infections [27]. STAT5 induced expression of CSF2 results in increased production of monocytic chemoattractant protein 1 (CCL2) by monocytic cells, which functions in the recruitment of monocytic cells, including macrophages to the sites of injury or infection [28]. Therefore, if *CSF2* is a *bona fide miR-147* target *in vivo* then a decrease in *miR-147* expression in PRRSV infected macrophages may act in the antiviral response by increasing CSF2 production. However, this may also result in the increased migration of macrophages to sites of PRRSV infection thus increasing host susceptibility.

To decipher the potential function(s) of *miR-147* down regulation in PRRSV-infected macrophages, transfection of a synthetic *miR-147* mimic was utilized to maintain *miR-147* in PAMs upon PRRSV infection. *MiR-147* levels were several hundred-fold higher in macrophages transfected with the *miR-147* mimic compared to macrophages transfected with the negative control mimic (Figure 5A). Increased *miR-147* levels in PRRSV-infected macrophages resulted in a significant decrease in PRRSV N protein mRNA levels at both 12 hpi and 24 hpi (Figure 6). This decrease in N protein expression translated to a small but noticeable reduction in PRRSV titers (Figure 7). These results imply that the decline of *miR-147* levels upon PRRSV infection may have some benefit for virus production. However, whether *miR-147* down-regulation is directly induced by PRRSV or if it is part of the cellular response to infection and PRRSV happens to benefit indirectly remains to be determined. A couple of recent studies have suggested a role for cellular miRNAs in PRRSV replication. For example, introduction of *miR-181* mimics into PAMs also reduced PRRSV replication *in vitro* [6]. It was suggested that this reduction is likely due to a *miR-181* seed match downstream of ORF4. PRRSV replication was also reduced in MARC-145 cells transfected with a *miR-125* mimic [7]. The reduction was attributed to increased NFκB due to the decreased expression of the negative regulator κB-RAS2, a *miR-125* target gene. Taken together, these studies, along with the work presented here, demonstrate complex multifaceted interactions exist between PRRSV and the cellular miRNA regulatory system.

Several large DNA viruses, particularly herpesviruses encode their own set of miRNAs [29]. Virally-encoded miRNAs are thought to be less important in RNA virus infections. This is likely to due to factors such as, genome size constraints, the potential for viral genome degradation, and precursor miRNA processing [30]. To ascertain the potential for PRRSV-encoded miRNAs sequencing reads which matched the PRRSV genome were analyzed for their ability to form the prototypical hairpin structure of pre-miRNAs. However, we could find no evidence of potential miRNA stem loop structures in the genomic context of these sequences in the PRRSV genome. This suggests that virally-encoded miRNAs are likely not an important aspect of PRRSV replication. However, the possibility of virally-encoded miRNAs expressed explicitly at time points other than those assayed cannot be discounted.

Generation of miRNA profiles of PRRSV infected alveolar macrophages has identified forty cellular miRNAs whose expression is significantly altered within the first forty-eight hours of PRRSV infection *in vitro*. This suggests that miRNAs are likely important mediators of PRRSV replication and host defense to infection. Target gene identification for selected miRNAs suggests that these miRNAs are involved in regulating immune signaling pathways, cytokine and transcription factor production. Forced over expression of *miR-147*, the most highly repressed cellular miRNA 24 hpi has a negative impact on PRRSV replication. Overall, the present study has revealed that a large and diverse group of miRNAs are expressed in swine alveolar macrophages and that the expression of a subset of these miRNAs is altered in PRRSV infected macrophages.

## Supporting Information

Table S1 Primers used in this study.
(XLSX)
